# Replacement of Albumin by Preovulatory Oviductal Fluid in Swim-Up Sperm Preparation Method Modifies Boar Sperm Parameters and Improves In Vitro Penetration of Oocytes

**DOI:** 10.3390/ani11051202

**Published:** 2021-04-22

**Authors:** Sergio Navarro-Serna, Evelyne París-Oller, Ondrej Simonik, Raquel Romar, Joaquín Gadea

**Affiliations:** 1 Department Physiology, International Excellence Campus for Higher Education and Research “Campus Mare Nostrum” and Institute for Biomedical Research of Murcia (IMIB-Arrixaca), University of Murcia, 30071 Murcia, Spain; sergio.navarro3@um.es (S.N.-S.); evelynemercedes.paris@um.es (E.P.-O.); ondrej.simonik@ibt.cas.cz (O.S.); 2 Laboratory of Reproductive Biology, Institute of Biotechnology of the Czech Academy of Sciences, BIOCEV, 252 50 Vestec, Czech Republic; 3 Department of Veterinary Sciences, Faculty of Agrobiology, Food and Natural Resources, Czech University of Life Sciences Prague, 165 00 Prague, Czech Republic

**Keywords:** oviductal fluid, swim-up, sperm selection, albumin, porcine

## Abstract

**Simple Summary:**

Part of the success of assisted reproductive techniques lies in gamete manipulation before the in vitro fertilization (IVF) procedure. Current chemically defined handling media lack crucial components for embryo development that exist under in vivo conditions. Recent studies of pigs have shown that the addition of reproductive fluids during in vitro fertilization and embryo culture improves embryo quality and their epigenetic profile in vitro. Porcine oviductal fluid (pOF) has been used to supplement the handling media used for sperm selection by centrifugation. However, its effect during sperm selection by the swim-up procedure is still unknown, as is the likely beneficial effect of replacing bovine serum albumin (BSA) by pOF as a protein source in sperm preparation media. In this study, four protein combinations in the swim-up medium were tested: 1 mg/mL BSA (the regular supplementation), 1% preovulatory pOF (1% pOF), 1 mg/mL BSA plus 1% pOF; and 5 mg/mL BSA. After sperm selection, various sperm parameters were assessed, and oocytes were inseminated in vitro. Results showed that the replacement of BSA by pOF improves some sperm motion parameters and increases in vitro oocyte penetration ability, whereas the combination of BSA + pOF did not show relevant effects. High concentrations of BSA had a detrimental effect, resulting in a decrease of sperm penetration.

**Abstract:**

More suitable and efficient methods to protect gametes from external harmful effects during in vitro handling can be achieved by adding preovulatory porcine oviductal fluid (pOF) to in vitro culture media. The objective of this study was to assess the swim-up procedure’s suitability as a sperm selection method using a medium supplemented with 1mg/mL BSA, 1% preovulatory pOF (*v*/*v*), 1% *v*/*v* pOF plus 1mg/mL BSA, and 5mg/mL BSA. After selection, various sperm parameters were studied, such as sperm recovery rate, sperm morphology, motility (by CASA), vitality, acrosome status and intracellular calcium (by flow cytometry) and ability to penetrate oocytes in vitro. Around 2% of sperm were recovered after swim-up, and the replacement of BSA by pOF showed a beneficial reduction of motility parameters calcium concentration, resulting in an increased penetration rate. The combination of albumin and oviductal fluid in the medium did not improve the sperm parameters results, whereas a high concentration of BSA increased sperm morphological abnormalities, motility, and acrosome damage, with a reduction of calcium concentration and penetration rate. In conclusion, the replacement of albumin by preovulatory oviductal fluid in the swim-up sperm preparation method modifies boar sperm parameters and improves the in vitro penetration of oocytes.

## 1. Introduction

After mating or artificial insemination in pigs, spermatozoa travel up the female reproductive tract from the cervix where they are initially deposited, up to the fertilization site at the oviduct [1]. During this journey, seminal plasma content is reduced, and a large number of spermatozoa die, with only a thousand or fewer reaching the fertilization site to fertilize the ovulated cumulus-enclosed oocytes [1]. Within the female tract, sperm cells interact with many different components found in the reproductive fluids [2]. Under in vitro conditions, an efficient sperm selection method must provide spermatozoa with adequate motility and morphology, maintaining high-quality spermatozoa’s viability by removing the toxic and lytic effect induced by abnormal and dead spermatozoa [3]. Procedures performed in the laboratory during gamete selection and manipulation impact embryo quality, i.e., sperm selection methods affect the capacitation status of spermatozoa, further impacting the fertilization process [4]. Finding more suitable and efficient methods to protect gametes from external harmful effects during handling can be achieved by mimicking in vivo conditions. However, regular in vitro procedures for sperm selection and capacitation do not recapitulate the in vivo sperm selection conditions, except in some complex devices using microfluidics [5]. In porcine species, many different protocols have been described for sperm selection (reviewed by Romar et al. 2019 [6]). Swim-up is a widespread sperm selection method used in human [7,8] and bovine species [9,10], consisting of adding a seminal sample at the bottom of a tube containing culture medium, allowing only motile sperm cells to travel up, and then being collected in the upper fraction at the top of the tube. Applying the swim-up technique to boar ejaculates confirmed that the selected populations were enriched for fast motile spermatozoa with normal morphology [11,12]. Moreover, when the swim-up method is performed with a medium formulated explicitly for boar semen, in vitro fertilization yield is improved compared to centrifugation with Percoll gradient [13]. So, sperm selection via the swim-up procedure is becoming a valid and promising method to be used in porcine in vitro fertilization studies [14,15,16].

In addition to the sperm selection method, the composition of capacitation and in vitro fertilization media is relevant, since it affects the fertilization process [17]. In this sense, bovine serum albumin (BSA) and fetal bovine serum are the most commonly used source of proteins in chemically defined media (reviewed by [18]). However, with the aim of reproducing the in vivo environment and allowing the beneficial interaction of sperm cells with molecules present in the oviductal secretions, several studies have enriched the sperm capacitation media with porcine oviductal fluid (pOF) (Table 1). pOF composition varies throughout the oestrus cycle [19], so considering that, naturally, the spermatozoa reach the fertilization site at the periovulatory stage, the pOF collected from animals at the late follicular phase is the one most frequently used to mimic in vivo conditions, as summarized in Table 1. The beneficial results of pOF observed in these studies slightly vary due to differences in experimental conditions, mainly the sperm preparation method and duration of sperm cells incubation with pOF.

Swim-up is a simple sperm procedure that takes around 20–30 min to be completed, meaning that sperm cells are in contact with the employed medium and its additives only for a short time. Despite this brief exposition, sperm motion parameters and capacitation status are affected, and substituting BSA by pOF might modulate sperm capacitation and prevent spontaneous acrosome reaction, as recently shown in bovine species [20]. However, the likely beneficial effects of replacing BSA for pOF in the swim-up procedure in pigs have not been studied yet, despite in vitro fertilization (IVF) efficiency in pigs still being low compared to other livestock species [6,18]. Therefore, the objective of this study is to explore whether different protein concentrations and combinations (BSA and pOF) in the swim-up medium further improve sperm quality parameters and whether this beneficial effect is further transferred to in vitro fertilization outcomes in a pOF-free IVF medium. This approach would allow the actual effect of pOF and swim-up procedure on further porcine IVF efficiency to be elucidated.

## 2. Materials and Methods

### 2.1. Ethics

The study was developed according to Spanish Policy for Animal Protection (RD 53/2013), which conforms to European Union Directive 2010/63/EU regarding the protection of animals used in scientific experiments. This project was authorized by the Ethical Committee at the University of Murcia and by the Animal Production Service of Agriculture Department of the Region of Murcia (Spain) (Ref number A13170705).

### 2.2. Sperm Preparation

All chemicals were purchased from Sigma-Aldrich Química, S.A. (Madrid, Spain) unless otherwise indicated.

Heterospermic samples of commercial porcine seminal doses for artificial insemination were supplied by AIM Ibérica (Calasparra, Murcia, Spain), stored at 16 °C and used within 1–3 days after ejaculation. Before swim-up, samples were centrifuged (1900 g, 15 min, at 21 °C in 50 mL tubes) to remove semen extender. Then, the pellet was resupended with a low volume of the semen extenderto concentrate cells to a range of 300–500 × 10^6^ spermatozoa/mL. After centrifugation, 1 mL swim-up medium (NaturARTs PIG sperm swim-up medium, Embryocloud, Murcia, Spain) supplemented with BSA, pOF (NaturARTs POF-LF, Embryocloud, Murcia, Spain) or both was added to a 10 mL conical tube and 1 mL semen was then placed at the bottom of the tube below the former layer. The tube was incubated at 38 °C with a 45° inclination for 20 min [13]. After incubation, 500 μL from the top of the conical tube were carefully collected and used to assess sperm quality parameters and IVF.

### 2.3. Evaluation of Sperm Functionality Parameters

Before sperm manipulation and after swim-up procedure with medium containing different protein supplementation, the following sperm quality and functionality parameters were analyzed.

#### 2.3.1. Sperm Concentration and Morphology

Sperm samples were fixed and diluted 1:10 (*v*/*v*) in saline with 0.3% formaldehyde. An improved Neubauer hemocytometer (HEMO, Paul Marienfeld GmbH & Co, Lauda-Königshofen, Germany) was used to evaluate sperm concentration by contrast phase microscopy at 200 × magnification (Leica DMR, Wetzlar, Germany), counting each sample in duplicate. Sperm recovery rate was calculated as the concentration of spermatozoa after swim-up divided by the concentration of spermatozoa before swim-up. This value was represented as a percentage.

To evaluate sperm morphology, a 10 μL-sample drop was placed on a slide, covered with a 24 mm × 24 mm cover slip and morphology were evaluated by contrast phase microscopy at 1000 × magnification (Leica DMR, Wetzlar, Germany). Two-hundred spermatozoa per sample were counted and classified into sperm with normal morphology, sperm with proximal cytoplasmatic droplets, sperm with distal cytoplasmatic droplets, sperm with folded tail, sperm with coiled tail and sperm with other abnormal morphologies [27].

#### 2.3.2. Analysis of Motion Parameters by CASA

Motion parameters were analyzed using a computer-assisted sperm analysis (CASA) system (ISAS V1, Proiser, Valencia, Spain) according to protocol previously described [28]. Before analysis, the chamber (20 μm depth; Spermtrack chamber, Proiser, Valencia, Spain) was warmed at 38 °C. A 4 μL-drop seminal sample was placed on the chamber and at least 3 fields and more than 200 cells of each sample were analyzed.

The CASA-derived motility parameters studied were progressive motility (%), curvilinear velocity (VCL, µm/s), straight-line velocity (VSL, µm/s), average path velocity (VAP, µm/s), linearity of the curvilinear trajectory (LIN, ratio of VSL/VCL, %), straightness (STR, ratio of VSL/VAP, %), wobble of the curvilinear trajectory (WOB, ratio of VAP/VCL, %), amplitude of lateral head displacement (ALH, µm) and beat cross-frequency (BCF, Hz) [3]. The setting parameters were 100 frames, in which spermatozoa had to be present in at least 15 frames to be counted, and 2 s with 50 images/second. Images were obtained using a negative contrast phase microscope at 100× magnification (Leica DMR, Wetzlar, Germany) and a digital camera (Basler Vision, Ahrensburg, Germany). Spermatozoa with a VAP < 10 mm/s were considered immotile [3].

#### 2.3.3. Sperm Viability and Acrosome Reaction

Sperm viability and acrosome integrity were simultaneously evaluated by flow cytometry using propidium iodide (PI) and fluorescein isothiocyanate-labeled peanut agglutinin (FITC-PNA) in Guava easyCyte^®^ (Merck Millipore, Madrid, Spain), with blue laser (488 nm) and FL1 sensor using 525 nm band-pass filter to detect FITC-PNA; and FL3 sensor using a 650 nm band-pass filter to detect PI, as previously described [20]. Staining stock solution was prepared by adding 10 mL Beltsville thawing solution (BTS) extender (Zoitech, Madrid, Spain), 50 µL PI 500µg/mL and 100 µL FITC-PNA (200 μg/mL). Samples were prepared by adding 500 µL staining stock solution and sperm with a final concentration between 1–2 × 10^5^ spermatozoa/mL. Samples were then incubated for 15 min at room temperature in the dark before measurement. To correct the spectral overlap, a process of fluorescence compensation was applied. After flow cytometer analysis, sperm cells were categorized into four groups according to their staining pattern: viable sperm with intact acrosome (PI and FITC-PNA negative), viable sperm with damaged acrosome (PI negative, FITC-PNA positive), non-viable sperm with damaged acrosome (PI and FITC-PNA positive); and non-viable sperm with intact acrosome (PI positive, FITC-PNA negative). The results showed the percentage of viable sperm with intact acrosome, viable sperm with damaged acrosome and total sperm with damaged acrosome (non-viable sperm with damaged acrosome plus viable sperm with damaged acrosome).

#### 2.3.4. Intracellular Sperm Calcium Concentration

Intracellular calcium concentration and sperm viability were simultaneously evaluated by flow cytometry using PI and Fluo-3 AM [29]. Sperm samples were evaluated in Guava easyCyte^®^ (Merck Millipore, Madrid, Spain) with blue laser (488 nm) and FL1 sensor using 525 nm band-pass filter to detect Fluo-3 AM; and FL3 sensor using a 650 nm band-pass filter to detect PI.

After a swim-up procedure with different protein sources, samples were diluted in staining stock solution prepared by diluting 50 μL PI (500 μg/mL) and 10 μL Fluo-3 AM (1 mM) in 10 mL NaturARTs PIG sperm swim-up medium 5 mg/mL BSA. Next, samples were prepared by adding 500 μL staining stock solution and sperm with a final concentration between 1–2 × 10^5^ spermatozoa/mL. These samples were incubated for 10 min at 38 °C before measurement. After analysis, four groups were detected: viable sperm with low calcium concentration (Fluo-3 AM and PI negative), viable sperm with high calcium concentration (PI negative, Fluo-3 AM positive), non-viable sperm with high calcium concentration (PI and Fluo-3 AM positive); and non-viable sperm with low calcium concentration (PI positive, Fluo-3 AM negative).

Data were represented as the percentage of viable spermatozoa with high calcium concentration, total spermatozoa with high calcium concentration and total viability.

### 2.4. In Vitro Zygote Production

#### 2.4.1. In Vitro Maturation of Cumulus-Oocyte Complexes (COCs)

As previously described [4], COCs were obtained by aspiration from ovarian follicles between 3–6 mm diameter from ovaries collected in the slaughterhouse from Landrace × Large-White prepubertal gilts. Ovaries were transported to the laboratory within 1 h in saline with 1 g/L kanamycin at 38 °C and then washed once in 0.04% cetrimide solution and twice in saline with 1 g/L kanamycin at 38 °C. Under the stereomicroscope, COCs were selected and washed in Dulbecco’s PBS (DPBS) supplemented with 1mg/mL polyvinyl alcohol (PVA) (DPBS-PVA) and then washed in preequilibrated NCSU37 medium [30] supplemented with 10% (*v*/*v*) porcine follicular fluid. After washing, groups of 50-55 COCs were cultured in 500 µL NCSU37 supplemented with 10% porcine follicular fluid, 1 mM dibutyryl cAMP, 10 UI/mL eCG and 10 UI/mL hCG for 20–22h (38.5 °C, 5% CO_2_, 20% O_2_; saturated humidity), followed by 20–22 h in NCSU37 without dibutyryl cAMP, eCG and hCG.

#### 2.4.2. In Vitro Fertilization (IVF)

After 40–44 h, in vitro matured COCs were partially decumulated by repeated pipetting with an automatic micropipette, washed in TALP medium as described in Matás et al. [4], and located in groups of 50–55 oocytes per well containing 250 μL TALP medium. Oocytes were then inseminated with spermatozoa selected with different swim-up medium supplementations. After sperm selection, spermatozoa were diluted in TALP medium, concentration adjusted to 5000 cells/mL, and each well inseminated with 250 μL TALP with spermatozoa. After insemination, gametes were co-incubated at 38.5 °C and 5% CO_2_ for 18–20 h. Then, putative zygotes were fixed in glutaraldehyde and stained in Hoechst 33,342, as previously described [31]. Penetration rate, mean number of spermatozoa per penetrated oocyte (S/O), mean number of spermatozoa bound to the zona pellucida of penetrated oocytes (S/ZP), male pronucleus formation rate (%PNM), monospermy rate and efficiency, calculated as the percentage of monospermy oocytes with respect to total inseminated and penetrated oocytes respectively, were evaluated under an epifluorescence microscope (Leica DM4000B LED, Germany).

### 2.5. Statistical Analysis

Data were shown as mean ± standard error of the mean (SEM) and analyzed by one-way analysis of variance (ANOVA); experimental group (protein addition) was the independent variable and replicates the covariant factor.

For sperm motility parameters, a cluster analysis was performed to identify sub-groups of spermatozoa within the sperm population [12,28,32]. Data sets were prepared by merging raw data files from each measured sperm sample (values for VAP, VSL, BCF and ALH) [32]. After cluster analysis, each spermatozoon was classified into one of four sperm sub-populations: slow velocity and non-progressive, medium velocity and non-progressive, medium velocity and progressive; and fast velocity and progressive. The relative frequency of each sub-population in each experimental group was compared by ANOVA. When ANOVA revealed significant differences (*p*-value < 0.05), values were compared by pairwise multiple comparison post hoc test (Tukey).

### 2.6. Experimental Design

**Figure 1:**
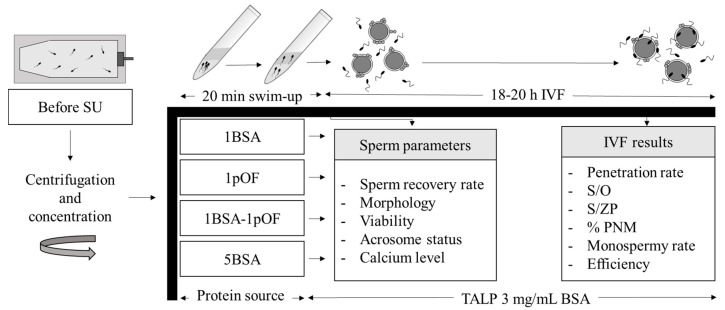
Schematic of the workflow procedure. Refrigerated ejaculated boar spermatozoa were centrifuged (Before swim-up group; SU) and then subjected to swim-up selection method in a medium supplemented with different protein combinations of bovine serum albumin (BSA group) and late follicular phase-porcine oviductal fluid (pOF): 1 mg/mL BSA (1BSA), 1% *v*/*v* pOF (1pOF group), 1 mg/mL BSA + 1% *v*/*v* pOF (1BSA-1pOF group) and 5 mg/mL BSA (5BSA group). Samples were incubated for 20 min at 38 °C with the tube inclinated 45° for swim-up selection. After selection, recovery rate, morphology, motility parameters, sperm viability, acrosome integrity, intracellular calcium concentrations and fertilization results after IVF in TALP medium supplemented with 3 mg/mL BSA were evaluated. S/O: number of spermatozoa per penetrated oocyte; S/ZP: number of spermatozoa bound to the zona pellucida of penetrated oocytes; PNM: male pronucleus formation rate.

Commercial swim-up medium was supplemented with different combinations of BSA and pOF as follows: 1 mg/mL BSA (1BSA); 1% *v*/*v* pOF (1pOF), 1 mg/mL BSA + 1% *v*/*v* pOF (1BSA-1pOF) or 5 mg/mL BSA (5BSA) (Figure 1). After sperm selection by swim-up, motility parameters, sperm recovery rate, morphology, viability, acrosome status, intracellular calcium concentration and IVF results were evaluated to determine the effect of each protein source in the swim-up medium. Furthermore, morphology, viability, acrosome status and intracellular calcium from seminal samples before centrifugation and sperm selection (before SU) were evaluated and compared with the other experimental groups. For gamete coincubation, TALP medium was supplemented with same protein and concentration in all groups, 3 mg/mL BSA, and no pOF was added. For sperm parameters, two analyses per sample were performed; and four replicates with 50–55 oocytes per group were preformed to evaluate IVF parameters.

## 3. Results

### 3.1. Sperm Recovery and Morphology

Sperm recovery rate after swim-up procedure ranged from 1.02–1.52% of the spermatozoa deposited in the bottom of the tube (Table 2). Medium containing 5 mg/mL BSA (5BSA group) led to a higher sperm recovery rate than 1pOF group, showing 1BSA and 1BSA-1pOF groups intermediate results.

Over 90% of sperm cells collected after swim-up showed normal morphology being this value higher than that observed in the sperm sample before manipulation and swim-up (group before SU) (Table 3). All swim-up treatments improved the morphology parameters compared to samples before swim-up, the 5BSA group being the one achieving the worst results among swim-up treatments (*p*-value < 0.01).

### 3.2. Sperm Motility, Motion Parameters (CASA), Sperm Viability and Acrosome Reaction

**Figure 2:**
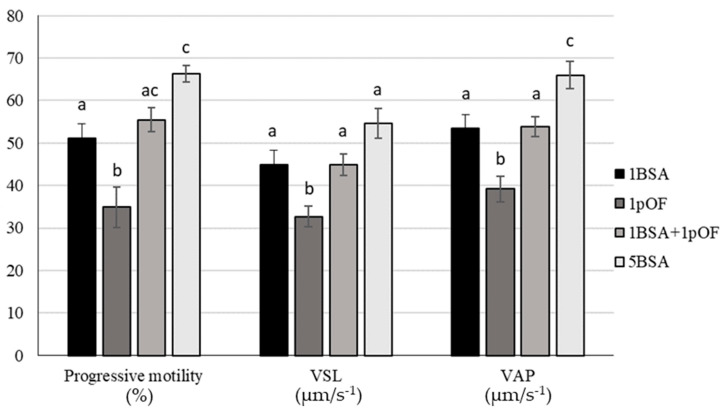
Motion parameters in ejaculated boar spermatozoa selected by swim-up method with different protein supplementation: 1 mg/mL BSA (1BSA), 1% *v*/*v* pOF (1pOF), 1 mg/mL BSA + 1% *v*/*v* pOF (1BSA-1pOF) and 5 mg/mL BSA (5BSA). Data were evaluated by computer-assisted semen analysis (CASA) and represented as mean ± SEM. ^a–c^ Values for each parameter are significantly different (*p*-value < 0.05). VSL; straight-line velocity; VAP, average path velocity.

Different protein source in swim-up medium affected the motility and motion parameters, except for LIN, STR and WOB (Table S1). When 5BSA concentration was used, motion parameters were higher than the 1pOF group, showing 1BSA and 1BSA-1pOF groups intermediate results (Table S1). As shown in Figure 2, 5BSA group reached the highest progressive motility and VAP results (*p*-value < 0.01), whereas 1pOF group showed the lowest value for straight-line velocity, VSL (*p*-value < 0.01).

In order to evaluate the presence and biological relevance of different sperm subpopulations according to their movement, cluster analysis was applied, resulting in the identification of 4 subpopulations named according to their motion parameters (Table S2). The proportions of spermatozoa within each sub-population after being selected with each swim-up medium were identified and differences between the distribution of subpopulations in each group analyzed (Table 4). Therefore, after the swim-up procedure, 5BSA group showed the highest percentage of spermatozoa with fast and progressive motility (*p*-value < 0.01). On the contrary, pOF had a slowing effect on sperm motility and semen samples subjected to swim-up supplemented, with 1% pOF (both 1pOF and 1BSA-1pOF groups) reaching the highest percentage of spermatozoa with slow non-progressive motility.

As for acrosome status and viability (Table 5), the percentage of spermatozoa viable with intact acrosome was higher after sperm selection by swim-up than before sperm selection (*p*-value < 0.01), but there were no differences among swim-up groups for this variable. Significant differences were found in viable cells with damaged acrosomes between groups supplemented with or without pOF (*p*-value < 0.01), but this difference might not have a biological relevance due to the low number of viable spermatozoa with damaged acrosomes.

### 3.3. Intracellular Sperm Calcium

Intracellular calcium measurements (Table 6) showed that 5BSA group had the lowest percentage of viable spermatozoa with high calcium concentration (*p*-value = 0.03). No differences were found on the percentage of spermatozoa with high calcium concentration nor percentage of viable spermatozoa.

### 3.4. In Vitro Fertilization Results

Sperm selected by different swim-up treatments were used to inseminate oocytes in a pOF-free IVF medium, where gametes were cocultured for 18–20 h. Results showed a lower ability of sperm selected by swim-up supplemented with 5 mg/mL BSA compared to other groups (Table 7). Interestingly, despite the fact that swim-up procedure took only 20 min, this time was enough to observe the beneficial effect of pOF on sperm functionality. Thus, swim-up medium supplemented with 1% pOF (alone or in combination with BSA) improved sperm penetration without increasing the mean number of sperm per penetrated. Moreover, 1pOF group reached similar monospermy rate and efficiency rate than 1BSA and 1BSA-1pOF groups, confirming the feasibility of replacing BSA by pOF in the boar sperm selection medium. Finally, no differences were found in male pronuclear formation rate, with all groups being over 95%.

## 4. Discussion

Current protocols used for porcine in vitro embryo production differ significantly from conditions that gametes, zygotes, and embryos encounter in vivo, resulting in a stressful environment for cells. Different studies clearly show that bringing both conditions closer together improves in vitro results [13,14,15,28,33,34]. The use of a swim-up method to select sperm, instead of protocols requiring density gradient centrifugations, is a clear example of such approximation. This procedure is routinely used in bovine and human seminal samples to isolate spermatozoa with high rates of normal morphology and motility rates and decreased DNA fragmentation [35,36]. In pigs, swim-up is an appropriate experimental system for sperm purification providing similarities with spermatozoa selected in vivo [12]. Moreover, in vitro embryos produced with swim-up sperm better resemble in vivo blastocyst in quality, expression, and methylation patterns [13]. However, unlike in other species, there is no standard culture protocol and medium to separate porcine sperm by swim-up, and different alternatives are employed [13,37]. Since proteins are a key media component due to their role in sperm capacitation and acrosome reaction, this study was focused on the effect on sperm characteristics after swim-up selection with a different protein source (albumin and pOF) and concentrations.

Selection of ejaculated boar sperm by swim-up for 20 min allowed a high sperm selection, as observed by the low recovery rate obtained (<2%), resulting in spermatozoa with higher rates of normal morphology, as previously reported [12]. By adding 5 mg/mL BSA, a slight improvement in the recovery rate and motility were achieved, but sperm morphology was impaired. It is well known that BSA mediates the efflux of sterols from the surface of spermatozoa, increasing membrane fluidity and allowing capacitation process under in vitro conditions [38,39]. The BSA’s role favoring sperm capacitation would explain the better sperm motility observed when BSA concentration was increased and, at the same time, the reason why fast, abnormal cells could reach the upper phase of the swim-up medium. Besides BSA’s role in sperm hyperactivation, the swim-up medium’s viscosity, when supplemented with a higher concentration of BSA (5 mg/mL), would explain the observed results since the addition of macromolecules increases the viscosity of the culture medium and, therefore, the sperm motility patterns. For instance, viscosity increase caused by methylcellulose’s addition improved boar sperm linearity and the proportion of spermatozoa with fast velocity and progressive motility [28]. In the current study, the likely increase in the medium viscosity due to the addition of biomacromolecules in a high concentration would support the better motility parameters and a higher proportion of spermatozoa with fast velocity and progressive motility in the 5BSA group. As expected, this favoring effect on motility and capacitation resulted in the highest rate of viable sperm with high calcium concentration in 5BSA group, but at the same time, had a detrimental effect on the ability of these spermatozoa to penetrate the oocytes, as shown by the lowest penetration rate reached in 5BSA group. After IVF, the penetration rate decreased as BSA concentration increased. As expected, the increase in penetration rate caused a decrease in monospermy rate, which agrees with the experiments previously reported [23]. The increase in penetration observed after selecting sperm with 1pOF versus 5BSA [13] or after pOF addition in IVF medium [40] has also been also reported. Together, these results would advise against the use of high concentrations of BSA to select spermatozoa to be used in porcine in vitro fertilization assays. Recently, Navarro-Serna et al. [41] employed a swim-up method and evaluated the penetration rate at different times, observing that the penetration rate increased from 4 to 9 h post insemination. We hypothesize that high concentrations of BSA might increase capacitation level in a short period, and therefore a lower percentage of spermatozoa would reach an optimal capacitation status at the fertilization time. So, the reported regulatory effect of oviductal fluid in the capacitation status [26] could allow many spermatozoa to reach a competent state to penetrate at the fertilization time and not before, which would not happen in BSA groups.

The replacement or combination of BSA by pOF might be a feasible option to modulate BSA’s immediate and evident effect on sperm hyperactivation even during a short contact period. We have previously shown that preovulatory pOF induces biochemical, biophysical, and functional changes in boar spermatozoa after a short incubation time of only 20 min [23], the same time that in our experimental conditions, the spermatozoa were incubated in the swim-up medium. These changes included a decrease in sperm motility parameters when spermatozoa were incubated with low molecular weight oviductal fluid proteins, and the increased numbers of spermatozoa with intact acrosomes [23]. Our results agree with those previously described, observing a moderation in the motility parameters in the sperm selected by swim-up supplemented only with 1% pOF compared to the groups containing BSA. We also found significant differences in the percentage of viable sperm with damaged acrosomes, being lower in those groups supplemented with pOF as described in previous studies [23,26], but with shallow numerical differences compared to those studies. The differences between the present and previously reported data might be based on different experimental conditions, since in these studies, spermatozoa were incubated in the presence or absence of pOF using Percoll gradient as the sperm selection method [23], or even no sperm selection at all [26].

Under our experimental conditions, no differences in motility parameters were observed when 1 mg/mL BSA was used alone (1BSA group) or combined with 1% pOF (1BSA-1pOF group). A modulation effect of preovulatory pOF on boar sperm capacitation is observed after 180 min incubation [26], but under our conditions, the rapid and robust capacitating effect of BSA could have masked the pOF effect. However, the slowing down of motility observed in pOF group did not have harm penetrability, since sperm prepared with pOF reached, bound, and penetrated the oocytes at the highest rate. However, this increase in sperm penetration was not translated into a higher final efficiency (putative zygotes obtained from total inseminated oocytes). It is important to highlight that the gamete coculture medium in the current study was not supplemented with pOF. Indeed, the current study’s objective was to assess the effect of pOF solely during sperm purification time. A long-time contact of gametes in the presence of pOF (18–20 h) might have improved the system’s final efficiency, as has been previously shown [13,34], since in these studies, monospermy rate and IVF efficiency were improved due to the combined effect of the incubation of sperm and oocytes with pOF.

## 5. Conclusions

In conclusion, using different protein sources during sperm selection by swim-up protocol affected sperm morphology, motility, viability, and capacitation parameters; and this effect was further transferred to fertilization results. Thus, the replacement of standard 1mg/mL BSA concentration with 1% *v*/*v* pOF reduces motion parameters without affecting viability and calcium homeostasis with a final improvement on sperm penetration. Combining BSA and pOF did not show significant improvements, whereas the addition of high BSA concentrations as 5mg/mL in the swim-up medium increased morpho-anomalies, premature capacitation, and decreased penetration rates, discouraging its use in porcine IVF protocols.

## Figures and Tables

**Table 1:** Effect of porcine oviductal fluid (pOF) from late follicular phase added to sperm incubation medium. Sperm incubation was done in presence of pOF under 38–39 °C and 5% CO_2_. F-T, frozen-thawed semen; ↑, increase; ↓, decrease; =, equal; pTyr, tyrosine phosphorylation; PKA, protein kinase A activity.

Boar Ejaculated Semen	pOF Concentration	Sperm Treatment	Main Results	Reference
Fresh	Pure, 1–5% (*v*/*v*)	Centrifugation followed by 1.5 h incubation	↑ sperm capacitation and prevents acrosome reaction	[21]
F-T	10 µg/mL glycoproteins	1 h incubation followed by centrifugation	= acrosome reaction (induced by calcium ionophore)	[22]
Fresh	50 µg/mL protein fraction (< or >100 kDa)	Percoll followed by 20 min incubation	↑ sperm viability, acrosome integrity and polyspermy ↓ sperm membrane fluidity	[23]
F-T	Pure, 20% (*v*/*v*)	Centrifugation followed by 6 h incubation	↑ pTyr	[24]
F-T	Pure, 20% (*v*/*v*)	Centrifugation followed by 1 h incubation	↑ pTyr, sperm intracellular calcium and motility	[25]
Fresh	Pure, 1% (*v*/*v*)	Swim-up for 20 min *	↑ IVF efficiency and improve epigenetic patterns **	[13]
Fresh	Pure, 1% (*v*/*v*)	3 h incubation	↓ pTyr, PKA and penetration rate	[26]

* pOF was present during swim-up procedure performed under 38.5 °C and air atmosphere. Gamete coincubation performed in medium containing pOF. ** pOF was present both during swim-up procedure and IVF.

**Table 2:** Ejaculated boar sperm recovery rate after sperm selection.

Group	*n*	Sperm Recovery Rate (%)
1BSA	16	1.11 ± 0.10 ^ab^
1pOF	16	1.02 ± 0.10 ^a^
1BSA-1pOF	16	1.20 ± 0.12 ^ab^
5BSA	16	1.52 ± 0.11 ^b^
*p*-value		0.01

Swim-up treatments were performed using different proteins sources and concentration: 1 mg/mL BSA (1BSA), 1% *v*/*v* pOF (1pOF), 1 mg/mL BSA + 1% *v*/*v* pOF (1BSA-1pOF) and 5 mg/mL BSA (5BSA). ^ab^ Values in the same column with different superscripts are significantly different (*p* < 0.05). *n*, number of analyzed samples.

**Table 3:** Ejaculated boar sperm morphology before treatment with swim-up (Before SU) and after sperm selection. Sperm morphology is expressed as mean ± SEM.

Group	*n*	Normal Morphology (%)	Bent Tails (%)	Distal Droplets (%)	Proximal Droplets (%)	Total Droplets (%)
Before SU	10	82.95 ± 1.47 ^a^	2.05 ± 0.65 ^a^	9.60 ± 0.99 ^a^	4.65 ± 0.44 ^a^	14.25 ± 1.12 ^a^
1BSA	10	98.50 ± 0.40 ^b^	0.40 ± 0.22 ^bc^	0.50 ± 0.22 ^b^	0.50 ± 0.22 ^b^	1.00 ± 0.21 ^b^
1pOF	10	97.10 ± 0.48 ^b^	0.50 ± 0.22 ^bc^	1.50 ± 0.40 ^b^	0.90 ± 0.31 ^b^	2.40 ± 0.56 ^b^
1BSA-1pOF	10	97.50 ± 0.45 ^b^	0.30 ± 0.21 ^c^	1.60 ± 0.37 ^b^	0.60 ± 0.22 ^b^	2.20 ± 0.30 ^b^
5BSA	10	93.70 ± 0.67 ^c^	1.30 ± 0.30 ^ab^	3.50 ± 0.58 ^c^	1.30 ± 0.34 ^b^	4.80 ± 0.65 ^c^
*p*-value		< 0.01	<0.01	<0.01	<0.01	<0.01

Swim-up treatments were performed using different proteins sources and concentration: 1 mg/mL BSA (1BSA), 1% *v*/*v* pOF (1pOF), 1 mg/mL BSA + 1% *v*/*v* pOF (1BSA-1pOF) and 5 mg/mL BSA (5BSA). ^a–c^ Values in the same column with different superscripts are significantly different (*p* < 0.05). *n*, number of analyzed samples.

**Table 4:** Cluster assay for motility of ejaculated boar spermatozoa selected by swim-up method with different protein supplementation. Cluster analysis is based on data from average path velocity (VAP), straight-line velocity (VSL), beat cross-frequency (BCF) and amplitude of lateral head displacement (ALH) obtained by computer-assisted semen analysis. Data expressed as mean ± SEM.

Group	*n*	Slow Velocity—Non-Progressive (%)	Medium Velocity—Non-Progressive (%)	Medium Velocity—Progressive (%)	Fast Velocity—Progressive (%)
1BSA	1188	36.53 ± 1.40 ^a^	30.05 ± 1.33 ^ab^	23.99 ± 1.24 ^a^	9.43 ± 0.85 ^a^
1pOF	2104	43.42 ± 1.48 ^b^	33.00 ± 1.40 ^a^	19.13 ± 1.17 ^b^	4.45 ± 0.62 ^b^
1BSA-1pOF	1124	39.83 ± 1.07 ^ab^	29.42 ± 0.99 ^b^	20.87 ± 0.89 ^ab^	9.89 ± 0.65 ^a^
5BSA	2329	36.11 ± 1.00 ^a^	28.30 ± 0.93 ^b^	21.47 ± 0.85 ^ab^	14.13 ± 0.72 ^c^
*p*-value		<0.01	0.01	0.04	<0.01

Swim-up treatments were performed using different proteins sources and concentration: 1 mg/mL BSA (1BSA), 1% *v*/*v* pOF (1pOF), 1 mg/mL BSA + 1% *v*/*v* pOF (1BSA-1pOF) and 5 mg/mL BSA (5BSA). ^a–c^ Values in the same column with different superscripts are significantly different (*p*-value <0.05). *n*, number of spermatozoa analyzed per group.

**Table 5:** Sperm viability and acrosome integrity of ejaculated boar sperm before treatment with swim-up (Before SU) and after sperm selection by swim-up.

Group	*n*	Viable Sperm with Intact Acrosome (%)	Viable Sperm with Damaged Acrosome (%)	Total Sperm with Acrosome Damaged (%)
Before SU	14	79.71 ± 1.32 ^a^	9.62 ± 1.13 ^a^	17.54 ± 1.62 ^a^
1BSA	14	89.34 ± 1.34 ^b^	0.74 ± 0.09 ^b^	3.61 ± 0.46 ^b^
1pOF	14	90.67 ± 0.92 ^b^	0.35 ± 0.04 ^c^	2.54 ± 0.31 ^b^
1BSA-1pOF	14	87.85 ± 1.18 ^b^	0.45 ± 0.11 ^c^	3.32 ± 0.33 ^b^
5BSA	14	87.06 ± 1.74 ^b^	0.89 ± 0.11 ^b^	4.16 ± 0.54 ^b^
*p*-value		<0.01	<0.01	<0.01

Swim-up treatments were performed using different proteins sources and concentration: 1 mg/mL BSA (1BSA), 1% *v*/*v* pOF (1pOF), 1 mg/mL BSA + 1% *v*/*v* pOF (1BSA-1pOF) and 5 mg/mL BSA (5BSA). ^a-c^ Values in the same column with different superscripts are significantly different (*p*-value <0.05). *n*, number of analyzed samples.

**Table 6:** Sperm viability and acrosome integrity of ejaculated boar sperm before treatment with swim-up (before SU) and after sperm selection by swim-up using different proteins sources and concentration. Calcium concentration and sperm viability were evaluated using flow cytometry after staining with Fluo-3 AM and propidium iodide. Data expressed as mean ± SEM.

Group	*n*	Viable Sperm with High Calcium Concentration (%)	Total Sperm with High Calcium Concentration (%)	Total Viable Sperm (%)
Before SU	10	28.88 ± 9.03 ^a^	55.72 ± 10.99	64.48 ± 2.45
1BSA	10	24.75 ± 2.36 ^a^	58.35 ± 6.32	59.57 ± 4.38
1pOF	10	30.24 ± 1.87 ^a^	54.99 ± 6.90	69.54 ± 5.72
1BSA-1pOF	10	27.76 ± 1.57 ^a^	52.62 ± 7.01	69.03 ± 7.18
5BSA	10	17.19 ± 1.15 ^b^	46.65 ± 7.92	60.91 ± 6.20
*p*-value		0.03	0.85	0.83

Swim-up treatments were performed using different proteins sources and concentration: 1 mg/mL BSA (1BSA), 1% *v*/*v* pOF (1pOF), 1 mg/mL BSA + 1% *v*/*v* pOF (1BSA-1pOF) and 5 mg/mL BSA (5BSA). ^ab^ Values in the same column with different superscripts are significantly different (*p* < 0.05). *n*, number of analyzed samples.

**Table 7:** IVF results after sperm selection by swim-up using different proteins sources and concentration. Four replicates were performed, and data are represented as mean ± SEM.

Group	*n*	Penetration (%)	S/O	S/ZP	PNM (%)	Monospermy (%)	Efficiency (%)
1BSA	178	74.16 ± 3.29 ^a^	2.65 ± 0.17 ^a^	11.28 ± 0.80 ^a^	99.42 ± 0.58	34.09 ± 4.14 ^ab^	25.28 ± 3.27
1pOF	189	86.77 ± 2.47 ^b^	2.49 ± 0.12 ^a^	9.60 ± 0.49 ^ab^	100	28.66 ± 3.54 ^a^	24.87 ± 3.15
1BSA-1pOF	197	76.65 ± 3.02 ^ab^	2.25 ± 0.10 ^ab^	7.69 ± 0.45 ^bc^	98.94 ± 0.75	31.79 ± 3.80 ^a^	24.37 ± 3.07
5BSA	199	59.30 ± 3.49 ^c^	1.92 ± 0.11 ^b^	6.19 ± 0.51 ^c^	100	48.31 ± 4.62 ^b^	28.64 ± 3.21
*p*-value		<0.01	<0.01	<0.01	0.312	<0.01	0.754

Swim-up treatments were performed using different proteins sources and concentration: 1 mg/mL BSA (1BSA), 1% *v*/*v* pOF (1pOF), 1 mg/mL BSA + 1% *v*/*v* pOF (1BSA-1pOF) and 5 mg/mL BSA (5BSA). ^a–c^ Values in the same column with different superscripts are significantly different (*p* < 0.05). *n*, number of oocytes evaluated; penetration rate (%), percentage of oocytes penetrated respect total oocytes; S/O, mean number of spermatozoa per penetrated oocyte; S/ZP, mean number of spermatozoa bound to the zona pellucida per penetrated oocyte; PNM (%), percentage of penetrated oocytes with male pronucleous formation; monospermy rate (%), percentage of monospermic zygotes respect to penetrated oocytes; efficiency (%), percentage of monospermic zygotes respect to total oocytes.

## Data Availability

The data presented in this study are available within the article.

## Supplementary Materials

The following are available online at https://www.mdpi.com/article/10.3390/ani11051202/s1, Table S1: Sperm motility before treatment with swim-up (Before SU) and after sperm selection by swim-up method with different protein supplementation, Table S2: Summary of sperm motility parameters derived from cluster analysis motility of spermatozoa selected by swim-up method with different protein supplementation.
