# Supplementary material for: Replacement of Albumin by Preovulatory Oviductal Fluid in Swim-Up Sperm Preparation Method Modifies Boar Sperm Parameters and Improves In Vitro Penetration of Oocytes
Source: Animals (Basel). 2021 Apr 22;11(5):1202. doi: 10.3390/ani11051202 (PMC8143553; doi:10.3390/ani11051202)
Supplement: Supplementary file 1 [file animals-11-01202-s001.zip › animals-1126977-supplementary.pdf]

Table S1. Sperm motility before treatment with swim-up (Before SU) and after sperm selection by swim-up method with different protein supplementation: 1 mg/mL BSA (1BSA), 1% v/v pOF (1pOF), 1 mg/mL BSA + 1% v/v pOF (1BSA-1pOF) and 5 mg/mL BSA (5BSA). Samples were measured by computer assisted semen analysis (CASA) and expressed as mean ± SEM. Curvilinear velocity (VCL, mm/s), straight-line velocity (VSL, mm/s), average path velocity (VAP, mm/s), linearity of the curvilinear trajectory (LIN, ratio of VSL/ VCL, %), straightness (STR, ratio of VSL/VAP, %), wobble of the curvilinear trajectory (WOB, ratio of VAP/VCL, %), amplitude of lateral head displacement (ALH, mm) and beat cross-frequency (BCF, Hz).

Group	N	Progressive motility (%)	VCL (μm s^{-1})	VSL (μm s^{-1})	VAP (μm s^{-1})	LIN (%)	STR (%)	WOB (%)	ALH (μm)	BCF (Hz)
Before SU	18	49.89 ±4.41 ^{a}	67.91 ± 2.99 ^{ab}	21.58 ± 1.51 ^{a}	38.80 ± 1.89 ^{a}	33.51 ± 1.86 ^{a}	55.51 ± 1.75 ^{a}	56.88 ± 1.38 ^{a}	2.81 ± 0.10 ^{ab}	6.57 ± 0.23 ^{ab}
1BSA	18	51.22 ±3.23 ^{a}	75.53 ± 3.63 ^{ac}	44.88 ± 3.47 ^{b}	53.44 ± 3.19 ^{b}	54.64 ± 2.93 ^{b}	75.00 ± 2.16 ^{b}	69.22 ± 2.10 ^{b}	2.92 ± 0.12 ^{ab}	6.47 ± 0.24 ^{ab}
1pOF	18	34.96 ±4.76 ^{b}	59.28 ± 5.40 ^{b}	32.71 ± 2.50 ^{c}	39.18 ± 3.09 ^{a}	54.66 ± 2.60 ^{b}	77.49 ± 1.76 ^{b}	67.05 ± 1.91 ^{b}	2.54 ± 0.21 ^{a}	5.86 ± 0.22 ^{a}
1BSA-pOF	18	55.50 ± 2.84 ^{ac}	75.34 ± 3.13 ^{ac}	44.86 ± 2.50 ^{b}	53.86 ± 2.30 ^{b}	55.18 ± 2.84 ^{b}	74.04 ± 2.43 ^{b}	69.88 ± 1.62 ^{b}	2.93 ± 0.14 ^{ab}	6.47 ± 0.14 ^{ab}
5BSA	18	66.39 ± 1.94 ^{c}	87.79 ± 3.08 ^{c}	54.62 ± 3.44 ^{b}	65.95 ± 3.21 ^{c}	56.98 ± 2.42 ^{b}	74.28 ± 2.00 ^{b}	72.98 ± 1.61 ^{b}	3.13 ± 0.09 ^{b}	7.18 ± 0.18 ^{b}
P-value		<0.01	<0.01	<0.01	<0.01	<0.01	<0.01	<0.01	0.05	<0.01

$^{a–c}$ Values in the same column with different superscripts are significantly different ($p < 0.05$). N, number of samples.

Table S2. Summary of sperm motility parameters derived from cluster analysis motility of spermatozoa selected by swim-up method with different protein supplementation: 1 mg/mL BSA (1BSA), 1% v/v pOF (1pOF), 1 mg/mL BSA + 1% v/v pOF (1BSA-1pOF) and 5 mg/mL BSA (5BSA). Cluster assay is based in VAP, VSL, BCF and ALH valued obtained by computer assisted semen analysis and expressed as mean ± SEM. Curvilinear velocity (VCL, mm/s), straight-line velocity (VSL, mm/s), average path velocity (VAP, mm/s), linearity of the curvilinear trajectory (LIN, ratio of VSL/ VCL, %), straightness (STR, ratio of VSL/VAP, %), wobble of the curvilinear trajectory (WOB, ratio of VAP/VCL, %), amplitude of lateral head displacement (ALH, mm) and beat cross-frequency (BCF, Hz).

Group	N	VCL (μm s^{-1})	VSL (μm s^{-1})	VAP (μm s^{-1})	LIN (%)	STR (%)	WOB (%)	ALH (μm)	BCF (Hz)
Slow no progressive	2601	43.12 ± 0.43 ^{a}	11.12 ± 0.14 ^{a}	22.33 ± 0.17 ^{a}	29.60 ± 0.41 ^{a}	50.56 ± 0.54 ^{a}	55.98 ± 0.34 ^{a}	2.17 ± 0.02 ^{a}	4.90 ± 0.04 ^{a}
Medium no progressive	2006	81.04 ± 0.67 ^{b}	33.75 ± 0.26 ^{b}	51.06 ± 0.27 ^{b}	47.83 ± 0.52 ^{b}	68.99 ± 0.57 ^{b}	67.38 ± 0.35 ^{b}	3.34 ± 0.03 ^{b}	7.31 ± 0.05 ^{b}
Medium progressive	1439	103.09 ± 0.62 ^{c}	66.12 ± 0.31 ^{c}	74.42 ± 0.29 ^{c}	67.18 ± 0.46 ^{c}	88.88 ± 0.36 ^{c}	74.38 ± 0.34 ^{c}	3.79 ± 0.03 ^{c}	8.37 ± 0.07 ^{c}
Fast progressive	699	130.18 ± 0.68 ^{d}	106.73 ± 0.58 ^{d}	107.63 ± 0.51 ^{d}	82.49 ± 0.39 ^{d}	97.09 ± 0.18 ^{d}	83.25 ± 0.31 ^{d}	3.94 ± 0.04 ^{d}	9.20 ± 0.09 ^{d}
P-value		<0.01	<0.01	<0.01	<0.01	<0.01	<0.01	<0.01	<0.01

$^{a–d}$ Values in the same column with different superscripts are significantly different ($p < 0.05$). N, number of spermatozoa analyzed per group.
